# Analysis of Residual Stress of Butt Fusion Joints for Polyethylene Gas Pipes

**DOI:** 10.3390/polym17101388

**Published:** 2025-05-18

**Authors:** Jie Gao, Minshuo Liang, Junqiang Wang, Sixi Zha, Ankang Yang, Huiqing Lan

**Affiliations:** 1 School of Mechanical, Electronic and Control Engineering, Beijing Jiaotong University, Beijing 100044, China; 2 China Special Equipment Inspection and Research Institute (CSEI), Beijing 100029, China; 3 School of Mechanical Engineering, Xinjiang University, Urumqi 830046, China

**Keywords:** polyethylene gas pipe, butt fusion joint, residual stress, non-uniform stress distribution, crystallinity

## Abstract

The performance of high-density polyethylene (PE) pipes joints directly affects the total pipeline’s operation, and so studying the residual stress of butt fusion joints is crucial for enhancing the safety of gas pipelines. Based on a layer-by-layer ring cutting test method, we measured the distribution of residual stresses in the fusion zone and heat-affected zone of butt fusion joints for PE gas pipes. Firstly, the ring samples were cut, their diameter changes were measured, and the results were compared with those predicted by the theoretical calculations. This showed that the circumferential residual stresses of the butt fusion joint for the PE gas pipes are exponentially distributed in the base material (BM) zone, the weld zone (WZ) and the heat-affected zone (HAZ). Furthermore, the residual stresses in the HAZ are lower than those in the BM zone, and the smallest residual stresses were seen in the WZ. Finally, using X-ray diffraction (XRD) technology, the crystallinities in the BM zone, the WZ, and the HAZ of the butt joints were measured. The crystallinity gradually decreased from the WZ to the HAZ and the BM zone, and the crystallinity in each zone was also related to the magnitude of the residual stresses.

## 1. Introduction

High-density polyethylene (HDPE) pipes are widely used in gas and water transmission because of their long service life, light weight, corrosion resistance, aging resistance, and superior impact resistance [1]. Compared with traditional metal pipes, HDPE pipes are flexible, easy to connect, and easy to construct, and these advantages make the number of gas leakage accidents that occur in HDPE gas pipes much lower than in steel pipes [2,3]. In the whole pipeline network system, the performance of the pipe joints directly determines the safety of pipeline operation, and the residual stress generated during the welding process of PE pipes is a key factor that affects the mechanical properties and service life of the butt fusion joints. Studies have shown that uneven heating and cooling during the butt fusion welding process can lead to complicated stress distributions in the weld zone of polyethylene (PE) pipes and may trigger problems such as stress corrosion cracking (SCC) [4,5]. Therefore, it is important to study the residual stress distribution in butt fusion joints of PE gas pipes to improve the safety of pipeline network operation [6].

Residual stress is the stress that remains inside the object after the removal of external load or a non-uniform temperature field. Such stresses are widely present in welding [7], heat treatment, and machining processes, and significantly affect the dimensional stability, damage resistance, and service life of the material [8]. Moslemi et al. [9] conducted a study on the welding residual stresses in AISI 316L stainless steel pipes and simulated the effects of different welding sequences by establishing a three-dimensional finite element model, and found that the choice of welding process plays an important role in the distribution of residual stresses. Distribution plays an important role in these processes. Kumar et al. [10] showed that the multi-pass welding of nickel-based alloy Inconel 617 could reduce the residual stress in the welded zone by lowering the heat input. Despite PE pipes differing from metals used in welding as non-metal materials, the method used to calculate the residual stress can be used for studying PE pipes. Biradar et al. [11] found that the peak stress in the welded joint of PE pipes could be significantly reduced by optimizing the heat input and annealing process. The optimization of the heat input and annealing process was found to significantly reduce the stress peaks in the joint area of PE pipes, thus improving the joint performance. The above studies provide useful references for understanding the formation mechanism of residual stresses in welded PE pipes, especially when analyzing the effects of different welding heat inputs on the stress distribution.

In the welding process of PE pipes, the distribution of residual stresses in the weld zone has a certain regularity. Wang [12] used finite element analysis (FEA) to simulate the four stages of HDPE pipe butt fusion welding. They found that the axial stress at the welded joint is low, while a significant difference is found in the distribution of circumferential stress on the inner and outer surfaces of the joint using thermal–force coupling finite element analysis, and at the same time, the radial stress is compressive stress at the rolled edge and tensile stress in the middle of the pipe wall. Their simulation results are basically consistent with the measured residual stresses. In addition, Sun et al. [13] further revealed the residual stress distribution law of the welded joints of HDPE pipes by combining the hole-drilling strain method and finite element simulation technology. It was shown that the annular residual stress was significantly higher than in the other directions and increased with the increase in pipe wall thickness. It is noteworthy that the maximum annular tensile stress is usually concentrated in the joint region near the outer wall, which is often a potential weak point of welded joints. This finding not only corresponds with the conclusion of Wang’s study but also further suggests that special attention should be paid to the stress optimization design in this field of practical engineering. Khademi-Zahedi et al. [14] further confirmed the prevalence of the annular stress concentration phenomenon through the study of repair patches and welded regions and suggested the use of PE100 material to alleviate the stress concentration in these regions.

The heat-affected zone (HAZ), as an important region in the welding process, has also received research attention to assess its residual stress distribution. During the welding process, the temperature in the HAZ is lower than the melting point of the material, but it is enough to cause changes in the microstructure of the material, which leads to its transformation from a semi-crystalline to an amorphous state [15]. These microstructural changes directly affect the hardness and toughness, as well as other mechanical properties, of the material in the HAZ, and play an important role in the distribution of the residual stresses. Xu et al. [16] pointed out that welding parameters such as preheating temperature and cooling rate have a more significant effect on the extent of the HAZ and the distribution of the residual stresses than others. It was found that a higher cooling rate reduces the extent of the HAZ and concentrates the peak distribution of the residual stresses. This is consistent with the results by Kogo et al. [17] for dissimilar materials welding, which found that the change in welding thermal gradient can significantly affect the stress distribution in the HAZ. Starostin et al. [18] further revealed the significant effect of low-temperature welding on the stress distribution in the HAZ, which showed that the low-temperature welding conditions did not significantly change the stress distribution pattern but the peak stress distribution was increased. Zhao et al. [19], undertaking X80 steel pipe ring weld joint research, showed that the multi-pass process and automated welding technology can effectively reduce the peak stress in the high-stress region of the weld zone (WZ). Although these studies were mainly on metallic materials, their conclusions are also applicable to the welding process of PE pipes, because the heat transfer characteristics and cooling rate of PE materials affect the residual stress distribution in a similar way.

Besides thermal gradients and molecular mobility, interfacial surface energy may also have a subtle influence on the welding behavior of PE. Although PE is chemically inert and exhibits low polar surface energy, van der Waals interactions such as London dispersion forces can still affect the degree of molecular entanglement and recrystallization at the weld interface [20]. While this study does not focus on surface energetics, such interfacial phenomena may potentially contribute to localized crystallinity variations and residual stress formation [21]. This perspective may help enrich the understanding of stress development mechanisms in polymer welding.

In recent years, the combination of numerical simulation techniques and experimental measurements has become the main means to study the residual stresses in welding. Perić et al. [22] used a combination of finite element simulation based on electron beam diffraction and experimental measurements to validate the accuracy of the residual stress distribution in the WZ of a thick-walled structure. Perić et al. [23] used X-ray diffraction to measure the residual stresses in the WZ of thick-walled pipes, and the experimental results were in good agreement with the numerical simulation results. Tan et al. [24] used the single-cut ring method and finite element simulation to further validate the reliability of the residual stress distribution law, and revealed the significant effect of the supplier’s production process on the distribution of the residual stresses. The above research method provides an important reference for the measurement of the welded residual stress distribution of PE pipes in this study.

Although the existing studies provide important theoretical references on the distribution law [25,26,27] and formation mechanism [28,29] of the welded residual stresses, systematic experimental validation studies are still scarce, especially for the experimental studies on the distribution law of residual stresses in the butt-fusion-welded joints and HAZs of PE gas pipes, which are even more scarce. In addition, the research on the relationship between the residual stress and material microstructure during the welding process is not yet sufficient to comprehensively reveal the formation mechanism of residual stress and its effect on the performance of welded joints. Therefore, in order to make up for these shortcomings, this paper carries out a systematic study on the residual stress distribution in butt fusion joints of PE gas pipes and the HAZs based on a layer-by-layer ring cutting test method. This is combined with theoretical calculations and X-ray diffraction technology. The butt fusion joint specimens analyzed in this paper are all welded to PE gas pipes according to a single high-pressure butt fusion process. Considering the temperature transfer and welding pressure during butt fusion welding, it is necessary to study the residual stress distribution, not only in the WZ but also in the pipe subjected to the secondary thermal effect near the joint, i.e., the residual stress distribution in the HAZ. Finally, the crystallinity of the WZ and the HAZ was determined by X-ray diffraction (XRD) techniques, which were used to analyze the reasons for the distribution of the residual stresses.

## 2. Method and Testing

### 2.1. Theory of Pure Bending Beams

**Figure 1:**
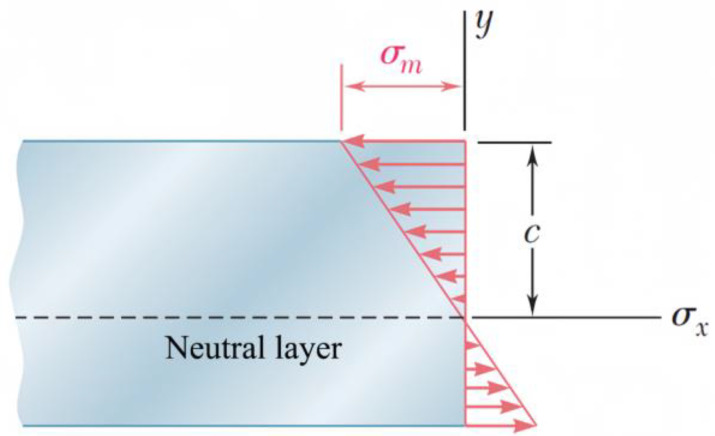
Linear distribution of uniaxial stress in the member.

In pure bending, the internal force on any cross-section of a symmetric member is equivalent to a couple, and the moment of the couple is called the bending moment at the cross-section. Assuming the material of the member is uniform, for the upper and lower surfaces of the member, *c* represents the maximum distance from the neutral layer (Figure 1). According to Hooke’s law for uniaxial stress state, within the elastic range, the normal stress varies linearly with the distance from the neutral layer [30].

When the distance *y* from the neutral layer reaches its maximum, the strain $\varepsilon_{x}$ also reaches its maximum:

(1) $$\varepsilon_{m}=\frac{c}{\rho }$$

In the case of pure bending, the centroid axis passes through the centroid of the cross-section, and can obtain the following:

(2) $$\sigma_{m}=\frac{Mc}{I}$$

where *I* is the moment of inertia of the cross-section around the centroid axis in the plane of the bending moment *M*.

Combining the Equations (1) and (2) gives

(3) $$\frac{1}{\rho }=\frac{M}{EI}$$

where *E* is the modulus of elasticity.

According to Equation (3), the deformation of the member caused by the bending moment *M* can be expressed in terms of the curvature of the neutral layer.

### 2.2. Layer-by-Layer Ring Cutting Test

The layer-by-layer ring cutting method has unique advantages over other residual stress measurement methods, such as hole drilling and X-ray diffraction. Hole drilling and X-ray diffraction usually only measure the residual stress distribution on the surface of the sample, and it is difficult to measure at different depths. Layer-by-layer ring cutting, on the other hand, can accurately measure residual stresses at different depths by removing material layer by layer. With each layer of material removed, the residual stress data of that layer can be obtained, thus providing a more comprehensive understanding of the stress state inside the sample. The layer-by-layer ring cutting method also provides a higher spatial resolution, making it particularly suitable for the residual stress analysis of thick-walled samples. For complex shapes or heterogeneous materials, traditional X-ray diffraction methods may cause measurement errors due to complex sample structures or material inhomogeneities. Layer-by-layer ring cutting overcomes these problems by removing layers one by one to accurately capture the residual stress distribution in each layer. In this experiment, the stability and accuracy of the measurement results of layer-by-layer ring cutting method were further verified by combining multiple experiments with data fitting.

**Figure 2:**
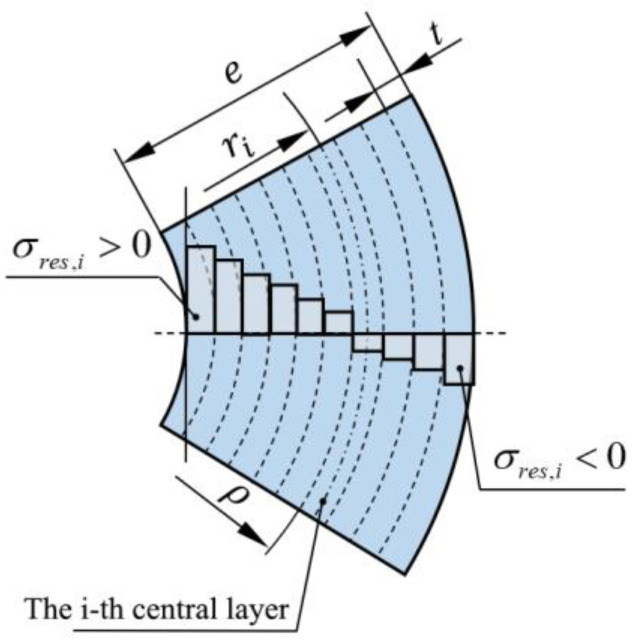
Schematic of sample layering and residual stress distribution.

The layer-by-layer ring cutting method involves dividing each pipe specimen into *k* layers along the wall thickness direction, with each layer having the same wall thickness and being considered as a bending beam, as shown in Figure 2. The radius of each layer will change due to the applied bending moment. Thus, the sum of the bending moment $M_{res,i}$ acting on each layer of the pipe wall is the total bending moment under the residual stress of the entire annular sample:

(4) $$M_{res}=\sum_{i=1}^{k}M_{res,i}$$

Assuming that each layer of the pipe wall has a constant circumferential residual stress $\sigma_{res,i}$, the bending moment $M_{res,i}$ of each layer of the pipe wall depends on the circumferential residual stress $\sigma_{res,i}$ in the layer. For samples where wall thickness is not removed, the bending moment can be expressed via the theory of bending beams:

(5) $$M_{res,i}=\sigma_{res,i}\cdot t\cdot w\cdot \left(\rho -r_{i}\right)$$

where *t* is the thickness of each layer of the pipe wall; t=e/k, *e* is the wall thickness of the pipe; *w* is the axial width of the annular sample; ρ is the radius of the neutral layer of the annular sample without removing the wall thickness; and $r_{i}$ is the central layer radius of the *i*-th layer.

The equilibrium condition for the circumferential residual stress of each layer of the pipe [31] is as follows:

(6) $$\sum_{i=1}^{k}\sigma_{res,i}=0$$

**Figure 3:**
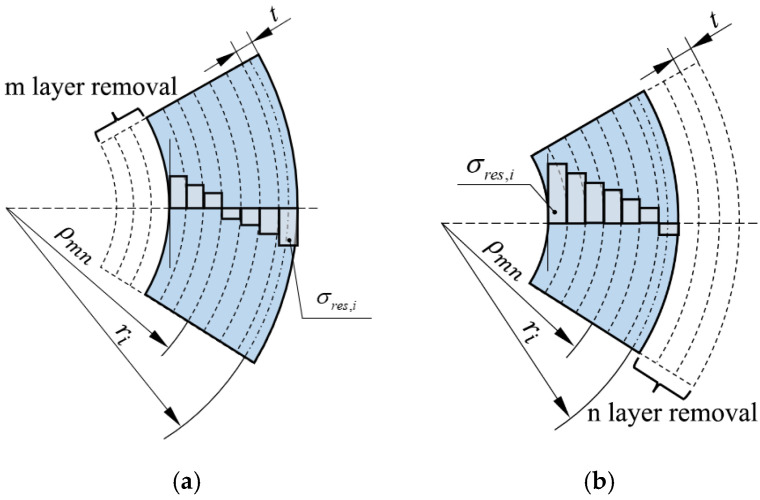
Schematic of annular sample with removed wall thickness. (**a**) Inner layer removal. (**b**) Outer layer removal.

For annular samples with *k* layers of pipe, we define the annular sample with different numbers of removed layers as $P_{mn}$, where *m* and *n* are the numbers of layers removed from the inner layer and the outer layer, respectively, as shown in Figure 3. In the annular sample with removed thickness, the remaining thickness’s residual stress bending moment $M_{res,i}^{mn}$ of each layer is expressed as follows:

(7) $$M_{res,i}^{mn}=\sigma_{res,i}\cdot t\cdot w\cdot \left(\rho_{mn}-r_{i}\right)$$

where $\rho_{mn}$ is the neutral layer radius of the annular sample $P_{mn}$ with different numbers of layers removed.

Therefore, the total residual stress bending moment of the remaining thickness of the sample with the inner layers removed is expressed as follows:

(8) $$M_{res}^{mn}=\sum_{i=m+1}^{k}M_{res,i}^{mn}$$

The total residual stress bending moment of the remaining thickness of the sample with the outer layers removed is expressed as follows:

(9) $$M_{res}^{mn}=\sum_{i=1}^{k-n}M_{res,i}^{mn}$$

**Figure 4:**
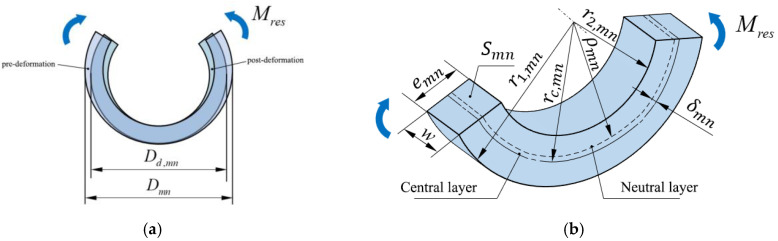
Schematic of annular sample deformation. (**a**) Deformation of annular sample under bending moment. (**b**) Annular sample cross-section.

Treating the entire annular sample as a bending beam, its radius changes due to the applied bending moment, as shown in Figure 4. The process of change in the outer diameter of the pipe annular sample under the bending moment $M_{res}^{mn}$ caused by the release of residual stress can be described by the following equation:

(10) $$\frac{1}{\rho_{d,mn}}-\frac{1}{\rho_{mn}}=\frac{M_{res}^{mn}}{E\cdot S_{mn}\cdot \delta_{mn}\cdot \rho_{mn}}$$

where $\rho_{d,mn}$ is the neutral layer radius of the ring after deformation; $\rho_{mn}$ is the neutral layer radius of the ring before deformation; *E* is the creep modulus; $S_{mn}$ is the cross-sectional area of the ring, $S_{mn}=w\cdot e_{mn}$; and $\delta_{mn}$ is the distance between the neutral layer and the center layer of the ring.

The bending moment $M_{res}^{mn}$ under the residual stress [32,33] is expressed as follows:

(11) $$M_{res}^{mn}=\frac{\left(\rho_{mn}-\rho_{d,mn}\right)\cdot E\cdot S_{mn}\cdot \delta_{mn}}{\rho_{d,mn}}$$

The radius of the neutral layer is as follows:

(12) $$\rho_{mn}=\frac{e_{mn}}{\ln\frac{r_{1,mn}}{r_{2,mn}}}$$

where $e_{mn}$ is the wall thickness of the ring; $r_{1,mn}$ is the outer radius of the ring; and $r_{2,mn}$ is the inner radius of the ring.

The distance between the neutral layer and the central layer of the ring is as follows:

(13) $$\delta_{mn}=r_{c,mn}-\rho_{mn}$$

where $r_{c,mn}$ is the radius of the center layer of the ring.

For a more intuitive expression of the diameter change of the annular sample under bending moment [34], the bending moment $M_{res}^{mn}$ after diameter change is expressed as follows:

(14) $$M_{res}^{mn}=\frac{\left(D_{d,mn}-D_{mn}\right)/2\cdot E\cdot S_{mn}\cdot \delta_{mn}}{\rho_{mn}-\left(D_{d,mn}-D_{mn}\right)/2}$$

where $D_{d,mn}$ is the diameter of the annular sample after deformation and $D_{mn}$ is the diameter of the annular sample before deformation.

Combining Equations (8), (9), and (14) with the equilibrium condition Equation (6) yields multiple linear equations that can be used to calculate the residual stress $\sigma_{res,i}$ of each layer for the pipe. By fitting the discrete residual stresses, the distribution of residual stress along the wall thickness direction can be obtained.

### 2.3. Pipe Base Material Test Scheme

The test material is a PE100 gas pipe (dn63, SDR11). The wall thickness of the PE gas pipe was reduced using a lathe. In the study, a high-precision CNC lathe is used to cut PE100 pipe samples with a water-based coolant to effectively reduce the thermal stresses and deformation generated during the cutting process. To minimize the introduction of mechanical stresses during the cutting process, we optimized the cutting speed to the range of 25–35 m/min and selected a feed pressure of 0.05–0.1 mm/rev. The selection of these parameters helps to minimize the excessive thermal and cutting forces that may be introduced by too high a cutting speed and too large a feed pressure, thus avoiding interference with the final residual stress measurement results. When cutting the samples, special attention is paid to reducing the thermal friction between the tool and the material, and the temperature rise in the cutting area is effectively reduced by using a water-based coolant, thus further minimizing the generation of thermal stresses and ensuring a more accurate residual stress measurement of the samples. At the same time, optimizing the cutting process and selecting the appropriate depth of cut contribute to reducing mechanical stress and ensuring the stability of the cut quality.

According to the equations in Section 2.2, the more layers the sample is divided into, the higher the accuracy of the residual stress fitting, resulting in a more accurate residual stress distribution. However, this requires higher precision in sample processing, increasing the workload. Here, the pipe was evenly divided into ten layers along the wall thickness direction, so the thickness removed for each layer was exactly one-tenth of the pipe wall thickness. In the layer-by-layer ring cutting method, the choice of axial length is crucial for ensuring the accuracy of the final residual stress measurement. According to Poduška et al. [35], the choice of axial length not only affects the structural integrity of the sample, but also directly relates to the distribution of residual stresses and the measurement results. Specifically, when the axial length is less than 5 mm, the gradient of residual stresses will be relatively small, which means that the axial residual stresses in the cut sample are almost negligible. Therefore, choosing 3 mm as the axial length can avoid additional stresses being introduced by excessively axial cuts and also maintain good sample stability to ensure that no unnecessary errors are generated during the measurement process due to excessive material size. The choice of 3 mm axial length is based on the precise control of material properties and stress distribution, which has been experimentally verified to effectively eliminate the influence of stresses introduced to the final test data during the cutting process. In addition, the size was chosen not only to optimize the stress state after cutting, but also to take into account the precision and handling of the experimental equipment in practice. In experiments, too large an axial length increases the risk of heat accumulation during the cutting process, which may introduce unnecessary residual stresses, which will directly affect the accuracy of the experimental results.

**Figure 5:**
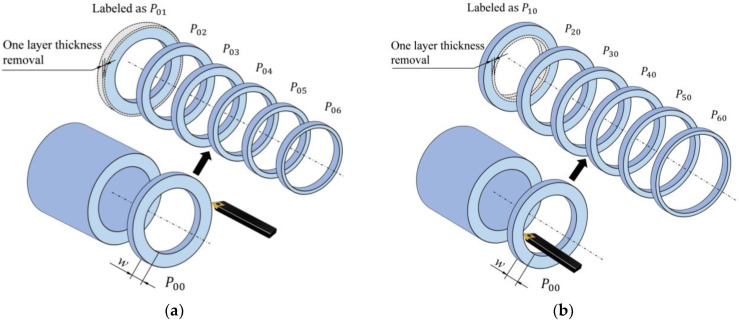
Removal samples: (**a**) outer layer removal samples; (**b**) inner layer removal samples.

The processed annular samples were labeled as $P_{mn}$, where *m* represents the number of layers removed from the inner layer, and *n* represents the number of layers removed from the outer layer. The annular samples without inner and outer layer removal were labeled as $P_{00}$. The annular samples with thickness removed layer by layer from the outer layer were numbered sequentially from $P_{01}$ to $P_{06}$ as shown in Figure 5a. Six annular samples were first produced, starting from the first sample, and one layer of wall thickness was gradually removed from the outer surface to obtain sample $P_{01}$. Two layers of wall thickness were removed to obtain the sample $P_{02}$, and so on, until the sixth sample was processed. The inner diameter of the annular samples remained unchanged, while the outer diameter decreased layer by layer.

The annular samples with thickness removed layer by layer from the inner layer were labeled sequentially from $P_{10}$ to $P_{60}$, as shown in Figure 5b. Similarly, six samples were produced, starting from the first sample, and one layer of wall thickness was gradually removed from the inner surface to obtain the sample $P_{10}$. Two layers of wall thickness were removed to obtain the sample $P_{20}$, and so on, until the sixth sample $P_{60}$ was processed. The outer diameter of the annular samples remained unchanged, while the inner diameter increased layer by layer.

**Figure 6:**
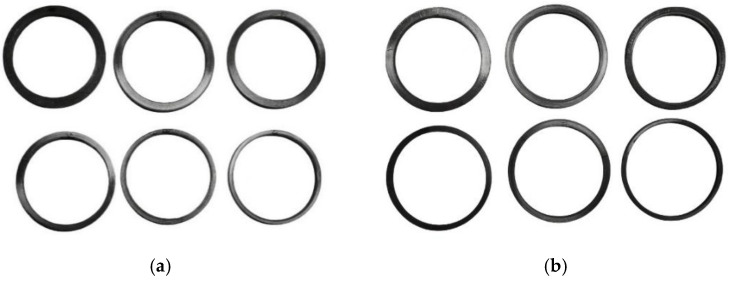
PE100 gas pipe BM annular samples: (**a**) outer cutting samples; (**b**) inner cutting samples.

During the machining of the pipe, the coolant was used to cool the samples during the cutting process to avoid the impact of cutting temperature on the samples and to minimize the generation of machining residual stress. The processed samples of PE100 gas pipe BM are shown in Figure 6. The completed samples were placed at a room temperature for over 6 h, and the outer diameter of each annular sample was measured and recorded one by one.

### 2.4. Circumferential Residual Stress Test for Pipe BM

**Figure 7:**
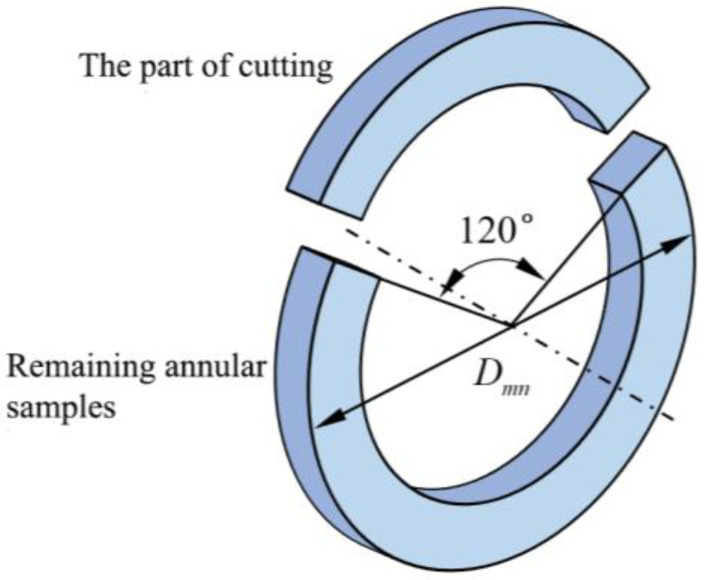
Partial cutting and outer diameter measurement of samples.

After obtaining 13 annular samples cut from the PE gas pipe, the annular part corresponding to the 120° central angle of each sample was axially cut to release the residual stress [35] in Figure 7. After cutting the corresponding annular part, the maximum diameter changes in the annular part occurred almost immediately after cutting. The diameter of the remaining annular samples was measured weekly.

**Figure 8:**
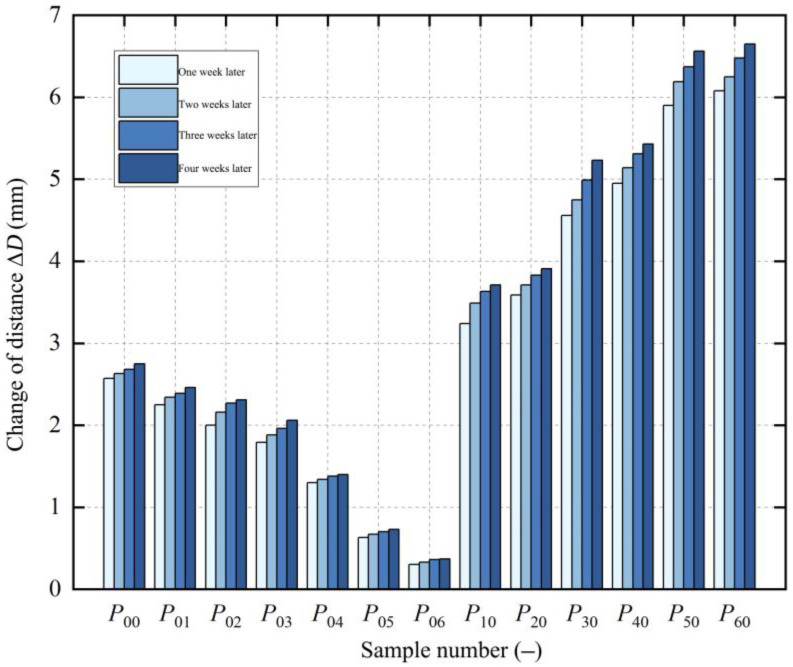
Diameter changes of BM samples over time.

After continuous measurements for four weeks, the diameter changes of each remaining annular sample are shown in Figure 8. However, due to the viscoelasticity of the PE material, the diameter changes continued to increase slightly over time. It was found that, after the fifth week, the changes in the diameter of the remaining annular samples decreased significantly. To save time, the paper used the diameter changes of the first four weeks to test the residual stress in the pipe.

According to Equations (7)–(9), the residual stress bending moment of the 13 samples after cutting are as follows:

(15) $$M_{res\;}^{mn}=\sum_{i=1}^{10}X_{i}^{mn}\cdot \sigma_{res,i}$$

where $X_{i}^{mn}$ is the residual stress coefficient for each layer.

By measuring the diameter changes of the 13 samples and using Equation (15), the residual stress bending moments for the 13 samples were obtained. Combined with the equilibrium condition in Equation (1), the overdetermined linear equation group was obtained:

(16) $$X_{RES}\cdot \sigma_{RES}=M_{RES}$$

In this linear equation system, the residual stress coefficient matrix $X_{RES}$ is as follows:

(17) $$X_{RES}=\left[\begin{array}{cccccccccc} X_{1}^{00} & X_{2}^{00} & X_{3}^{00} & X_{4}^{00} & X_{5}^{00} & X_{6}^{00} & X_{7}^{00} & X_{8}^{00} & X_{9}^{00} & X_{10}^{00}\\ X_{1}^{01} & X_{2}^{01} & X_{3}^{01} & X_{4}^{01} & X_{5}^{01} & X_{6}^{01} & X_{7}^{01} & X_{8}^{01} & X_{9}^{01} & 0\\ X_{1}^{02} & X_{2}^{02} & X_{3}^{02} & X_{4}^{02} & X_{5}^{02} & X_{6}^{02} & X_{7}^{02} & X_{8}^{02} & 0 & 0\\ X_{1}^{03} & X_{2}^{03} & X_{3}^{03} & X_{4}^{03} & X_{5}^{03} & X_{6}^{03} & X_{7}^{03} & 0 & 0 & 0\\ X_{1}^{04} & X_{2}^{04} & X_{3}^{04} & X_{4}^{04} & X_{5}^{04} & X_{6}^{04} & 0 & 0 & 0 & 0\\ X_{1}^{05} & X_{2}^{05} & X_{3}^{05} & X_{4}^{05} & X_{5}^{05} & 0 & 0 & 0 & 0 & 0\\ X_{1}^{06} & X_{2}^{06} & X_{3}^{06} & X_{4}^{06} & 0 & 0 & 0 & 0 & 0 & 0\\ 0 & X_{2}^{10} & X_{3}^{10} & X_{4}^{10} & X_{5}^{10} & X_{6}^{10} & X_{7}^{10} & X_{8}^{10} & X_{9}^{10} & X_{10}^{10}\\ 0 & 0 & X_{3}^{20} & X_{4}^{20} & X_{5}^{20} & X_{6}^{20} & X_{7}^{20} & X_{8}^{20} & X_{9}^{20} & X_{10}^{20}\\ 0 & 0 & 0 & X_{4}^{30} & X_{5}^{30} & X_{6}^{30} & X_{7}^{30} & X_{8}^{30} & X_{9}^{30} & X_{10}^{30}\\ 0 & 0 & 0 & 0 & X_{5}^{40} & X_{6}^{40} & X_{7}^{40} & X_{8}^{40} & X_{9}^{40} & X_{10}^{40}\\ 0 & 0 & 0 & 0 & 0 & X_{6}^{50} & X_{7}^{50} & X_{8}^{50} & X_{9}^{50} & X_{10}^{50}\\ 0 & 0 & 0 & 0 & 0 & 0 & X_{7}^{60} & X_{8}^{60} & X_{9}^{60} & X_{10}^{60}\\ 1 & 1 & 1 & 1 & 1 & 1 & 1 & 1 & 1 & 1 \end{array}\right]$$

The residual stress matrix $\sigma_{RES}$ is as follows:

(18) $$\sigma_{RES}={\left[\begin{array}{lll} \sigma_{res,1} & \ldots & \sigma_{res,10} \end{array}\right]}^{T}$$

The residual stress bending moment matrix $M_{RES}$ is as follows:

(19) $$M_{RES}={\left[\begin{array}{llllllll} M_{res\;}^{00} & M_{res\;}^{01} & \ldots & M_{res\;}^{06} & M_{res\;}^{10} & \ldots & M_{res\;}^{60} & 1 \end{array}\right]}^{T}$$

To solve for the residual stress values $\sigma_{res,i}$ of each layer, a corresponding MATLAB 2021 program was written to calculate the residual stress coefficient matrix $X_{RES}$ and the residual stress bending moment matrix $M_{RES}$. By performing the numerical solution of Equation (16), the residual stress values of each layer were obtained, and the discrete residual stress of each layer was further fitted to obtain the distribution curve of residual stress along the pipe wall thickness direction.

### 2.5. Distribution of Circumferential Residual Stress in Base Material

To more accurately fit the residual stress of each layer, normalization was performed along the wall thickness position:

(20) $$x_{r}=\frac{r_{i}-r_{2,00}}{e}$$

where $x_{r}$ is the relative position along the wall thickness, $r_{2,00}$ is the inner wall radius of the sample $P_{00}$ without removing wall thickness, and *e* is the wall thickness of the pipe and the wall thickness of the sample $P_{00}$.

According to the research of Poduška et al. [36], the circumferential residual stress distribution inside the PE pipe exhibits an exponential form, which is related to the exponential nature of the temperature gradient during the production extrusion cooling process. This can be fitted through exponential relationship as follows:

(21) $$\sigma_{res}(x_{r})=c_{1}+c_{2}e^{3.2x_{r}}$$

where $c_{1}$ and $c_{2}$ are coefficients that determine the magnitude of the residual stress, obtained by fitting the discrete residual stresses.

**Figure 9:**
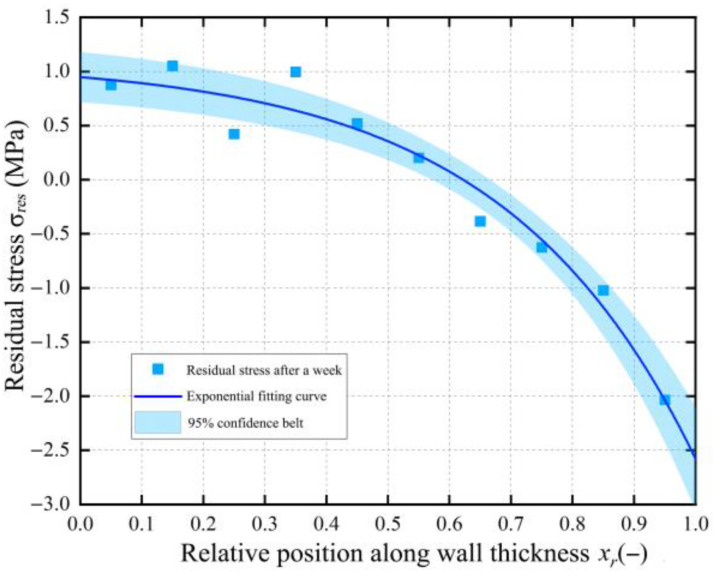
Distribution of circumferential residual stress in base material after one week.

The results of solving the residual stress in the BM pipe wall show that after one week of diameter changes, the distribution of circumferential residual stress released by the BM is $\sigma_{res}\left(x_{r}\right)=1.09808-0.14997\cdot e^{3.2x_{r}}$. With an R-squared value of 0.953 for the exponential fit, this can accurately describe the distribution of circumferential residual stress $\sigma_{res}$ in the BM pipe wall. The circumferential residual stress in the BM pipe exhibits a nonlinear distribution controlled by the exponential function $e^{3.2x_{r}}$, as shown in Figure 9. When $x_{r}=0$ at the inner wall position of the BM pipe, the residual stress value is 0.95 MPa; when $x_{r}=1$ at the outer wall position of the BM pipe, the residual stress value is 2.57 MPa. Here, the positive and negative signs represent the direction of the residual stress, indicating that the directions of the residual stresses near the inner and outer walls of the pipe are opposite.

## 3. Analysis and Discussion

### 3.1. Division of the HAZ of Butt Fusion Joints

During the butt fusion process, when the two pipes are butt-fusion-welded, assuming that the heat effect and welding pressure on them are equivalent, the performance of the butt fusion joints is symmetrically distributed along the weld seam. Therefore, only half of the joint is selected for the study of the residual stress in the butt fusion joints and their HAZ. By measuring the temperature, the temperature distribution of the pipe during the butt fusion process can be visually displayed. A thermal imaging thermometer is used to measure the temperature of the pipe to judge the heat transfer distribution during the pipe welding process and then determine the approximate range of the HAZ [37,38].

**Figure 10:**
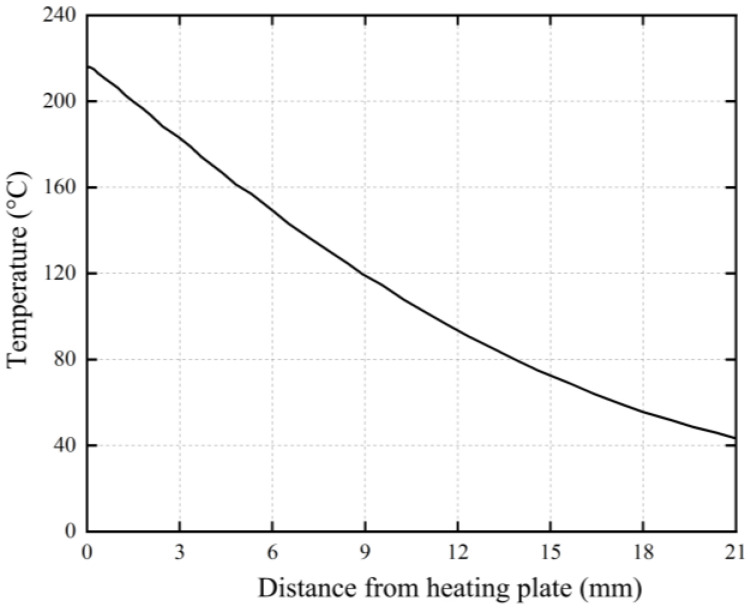
Temperature distribution of pipe on one end of heating plate.

PE pipes were welded by using a single high-pressure butt fusion process. At the end of the heat absorption time when the PE pipe was in contact with the heating plate, the outer surface temperature of the pipe on the side of the heating plate was measured along the axial direction of the pipe. The measured temperature distribution is shown in Figure 10. Considering that the axial width of the residual stress sample is 3 mm, the analysis is conducted at intervals of 3 mm. The melting temperature range of HDPE is approximately between 120 °C and 140 °C. The pipe material at a distance of 0~9 mm from the heating plate is in a molten state, where the region 0~3 mm from the heating plate is the initial curling edge generation area and the pipe material at 18~21 mm from the heating plate has a lower temperature, and so the region 9~18 mm from the heating plate is tentatively designated as the HAZ.

The welded fusion joint was cut from the weld seam. The pipe material 0~3 mm from the weld seam corresponds to that in the WZ 3~6 mm from the heating plate during welding. The pipe material 6~15 mm from the weld seam corresponds to that in the HAZ 9~18 mm from the heating plate during welding. The crystallinities of the pipe materials in the WZ, HAZ, and BM were measured, respectively.

The samples were tested by using the Bruker D8 ADVANCE X-ray diffractometer (Bruker AXS GmbH, Karlsruhe, Germany) with the test current of 40 mA, the voltage of 40 kV, the scanning angle of 5~90°, and the scanning rate of 5°/min. The Bruker TOPAS 6 software was used for crystallinity analysis. Through the function fitting method, the crystallinity $X_{c}$ was obtained by fitting the samples with the following formula:

(22) $$X_{c}=\frac{W_{c}}{W_{c}+W_{a}}$$

where $W_{c}$ is the area of crystalline peaks, and $W_{a}$ is the area of amorphous scatterings.

**Figure 11:**
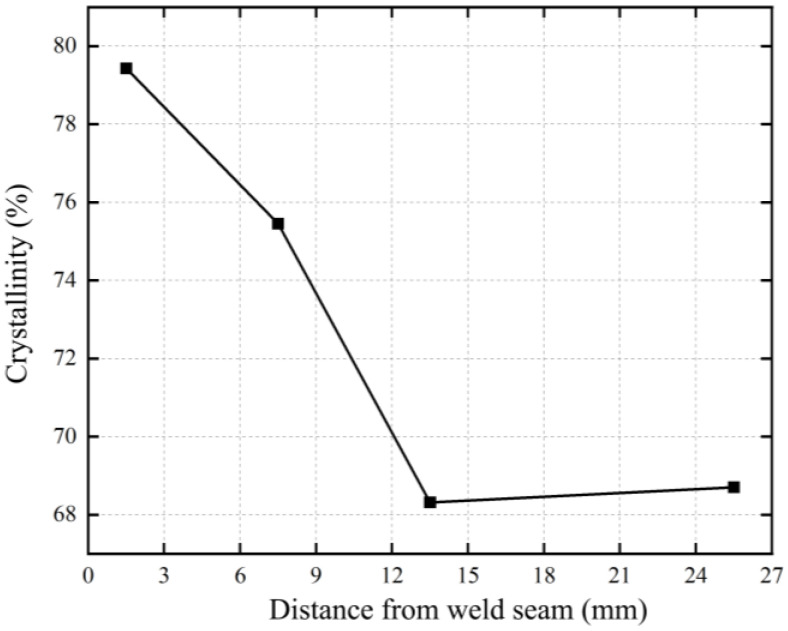
Crystallinity distribution of pipe on one end of weld.

The crystallinity obtained in this way is relative. To reduce errors, repeated fitting was used to minimize errors in the experiment. The crystallinity distribution at corresponding positions from the weld seam was calculated after repeated fitting, as shown in Figure 11. The material near the weld seam in the WZ has the highest crystallinity. There are significant differences in crystallinity between the HAZ at 6~9 mm and 12~15 mm from the weld seam, while the crystallinity of the material at 12~15 mm from the weld seam is not much different from that of the unaffected pipe material. It can be inferred that the boundary of the HAZ is 15~24 mm from the weld seam.

In Figure 11, it can be observed that the crystallinity decreases with increasing distance from the weld seam. This can be attributed to the temperature distribution and cooling rate during welding. Near the weld seam, the temperature is high, allowing for the formation of a more ordered crystalline structure as the material cools. In contrast, as the distance from the weld seam increases, the cooling rate decreases, leading to less ordered crystalline structures and lower crystallinity. The temperature gradient in the HAZ results in non-uniform cooling, which restricts molecular chain mobility and crystal growth, especially between 6 and 15 mm from the weld seam. Beyond 15 mm, the material temperature stabilizes, and the crystallinity approaches that of the unaffected base material.

Therefore, after combining the temperature distribution during the butt fusion process and the crystallinity distribution of the pipe material on one end of the fusion joint weld seam, the pipe material 3~15 mm from the weld seam in the HAZ was selected to study the residual stress distribution.

**Figure 12:**
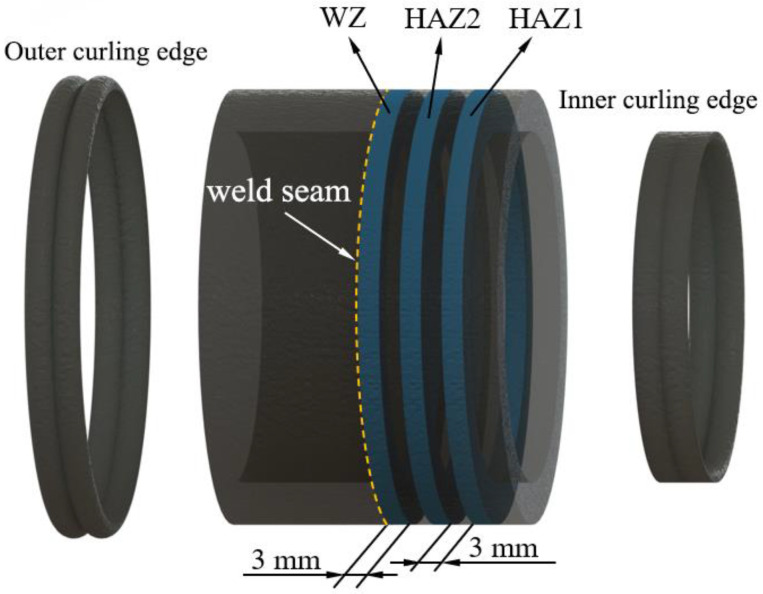
Selection of annular samples from butt fusion joints.

Similar to the BM test, the wall thickness of the joint samples was evenly divided into ten layers. Therefore, 13 butt fusion joint samples with the same process parameters were butt-fusion-welded for residual stress testing and analysis. As shown in Figure 12, to avoid the influence of the butt fusion curling edge on the diameter measurement and overall residual stress in the WZ, the inner and outer curling edges of each butt fusion joint sample were removed. This standardizes the inner and outer diameters and wall thicknesses of the annular samples, facilitating subsequent comparisons of the residual stress distribution in different zones. The samples were divided into the WZ samples, heat-affected zone 1 (HAZ1) samples and heat-affected zone 2 (HAZ2) samples. The axial thickness of these samples was 3 mm, consistent with the BM samples. The one end of WZ sample was adjacent to the weld seam, representing the WZ oft the pipe most affected by heat. Axially, HAZ1 was 3 mm away from the WZ, and HAZ2 was another 3 mm away from HAZ1. Next, according to the method in Section 3, the sample was processed by gradually removing the wall thickness layer by layer. For example, for the first joint sample, three annular samples without wall thickness removal were cut out from different regions, respectively. For convenient machining, the removal thickness of these three regions’ samples was kept the same when sampling each joint. WZ, HAZ1, and HAZ2 samples were cut from the 13 joint samples, respectively.

### 3.2. Residual Stress Testing of the Joints

**Figure 13:**
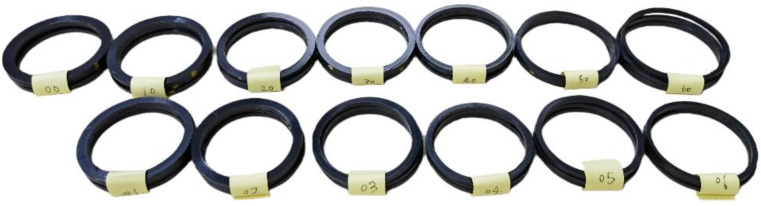
Annular samples from three zones of butt fusion joints.

During machining, the coolant was also used to cool the samples. A total of 39 annular samples were processed in Figure 13. The samples were left at room temperature for more than 6 h. Each sample was marked similarly to the BM samples, and the outer diameters were measured and recorded one by one.

**Figure 14:**
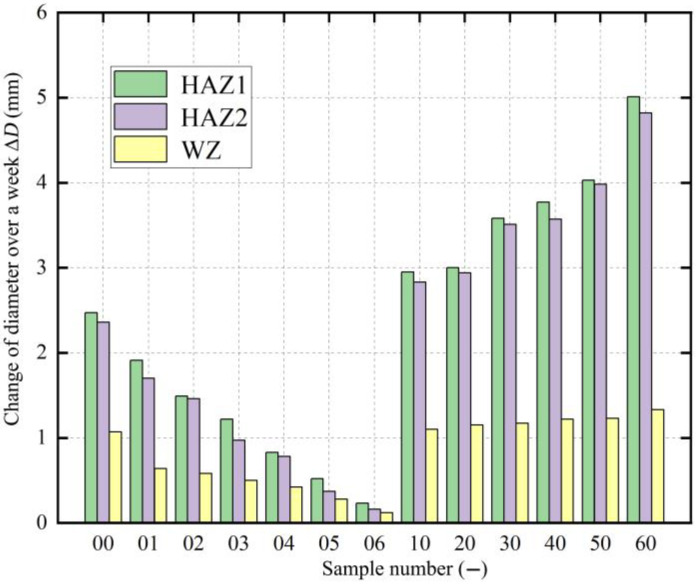
Diameter change of samples in three zones after one week.

After continuous measurements for four weeks, the diameter change trend of the samples was similar to that of the BM samples, with the diameter change gradually increasing each week. Figure 14 displays the diameter change of each remaining annular sample in the three zones after one week. The diameter was measured every other week, and the trend of change in each different zone was essentially the same.

From the overall change trend of all samples, the diameter changes in all the zones increased over time, similar to the BM samples. This is due to the material gradually stabilizing and the release of residual stress. The diameter change of HAZ1 samples was the largest, while that of HAZ2 samples was slightly smaller compared to HAZ1, reflecting the different degrees of the heat effect in the two zones during fusion. However, the difference in diameter change between HAZ1 and HAZ2 samples fluctuated, likely due to slight differences in the process parameters controlled during the fusion of each joint sample. The diameter change of the WZ samples was significantly different from that of the HAZ, with the smaller diameter change of WZ samples. This requires further analysis, combined with the establishment of the residual stress distribution in each zone.

### 3.3. Analysis of Circumferential Residual Stress Distribution at the Joint

**Figure 15:**
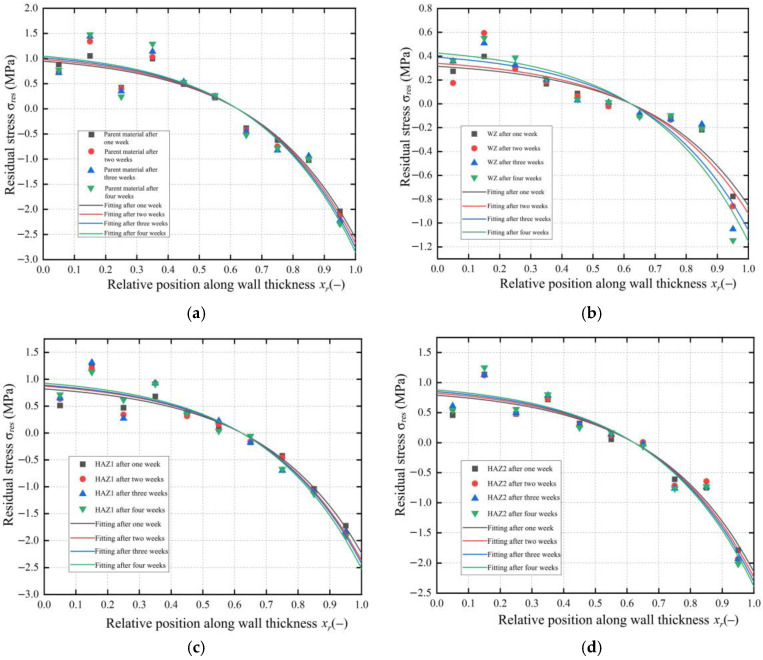
Residual stress distribution of the joint in different zones. (**a**) BM. (**b**) WZ. (**c**) HAZ1. (**d**) HAZ2.

Based on the residual stress method described in Section 3, the discrete values of residual stress for each wall thickness layer in the BM, WZ, HAZ1, and HAZ2 were calculated. Then, the discrete values were fitted exponentially to obtain the residual stress distribution along the wall thickness direction in the WZ, HAZ1, and HAZ2 of the joints after one week, two weeks, three weeks, and four weeks, respectively. The fitted residual stress distribution was used to evaluate the residual stress near the inner and outer walls of the pipe in WZ, HAZ1, and HAZ2 and compared with that in the BM in Figure 15. Figure 15a shows that the residual stress at the inner wall of the BM pipe is 1.05 MPa, and that at the outer wall is 2.75 MPa. The residual stress values at the inner and outer walls of the BM pipe are relatively large, obviously indicating the presence of initial residual stress in the PE pipe during production.

After butt fusion for the BM, the WZ and HAZ are generated and the residual stress levels and change trend differences in the different zones are found to be related to the material’s heat treatment history, cooling rate, material properties, and other factors. Figure 15b–d show the residual stress distribution in the WZ, HAZ1, and HAZ2. The residual stress distributions in these three zones can still be exponentially fitted using Equation (21). This indicates that just as the different cooling rates of the inner and outer walls during the production of PE BM pipe can lead to generate the residual stress, the changes in the residual stress in the different zones during butt fusion are similarly dominated by the cooling rates of the inner and outer walls.

Table 1 shows residual stress values on the inner and outer walls of each zone. The comparison reveals that the residual stresses on the inner and outer walls in the HAZ1 and HAZ2 are not significantly different, indicating that the degree of influence from the heat source was similar for these two regions during butt fusion. Compared to the BM, the residual stress in these two HAZs was improved to varying degrees, suggesting HAZ1 and HAZ2 were affected by the conduction of heat during butt fusion, which was the equivalent of the annealing process and eliminated some residual stress. The fluctuation of the residual stress on the inner and outer pipe walls of the WZ is minimal. During the fusion process, the WZ transitioned from the BM to a molten state and then recrystallized. During pressure holding and cooling, the PE molecular chains were interwoven more fully, and curling edge provided protection. This resulted in a more uniform cooling rate in the WZ compared to other zones, leading to a higher crystallinity compared to the BM. Therefore, the residual stress in the WZ is lower and is reduced more significantly compared to the BM zone. Overall, the residual stress levels observed in all regions are relatively low, as supported by the data in Table 1.

**Figure 16:**
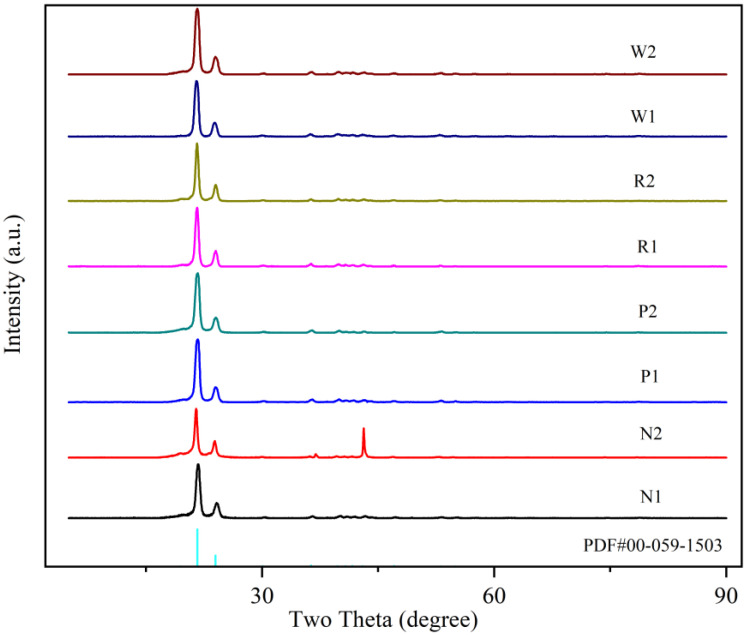
XRD patterns.

**Figure 17:**
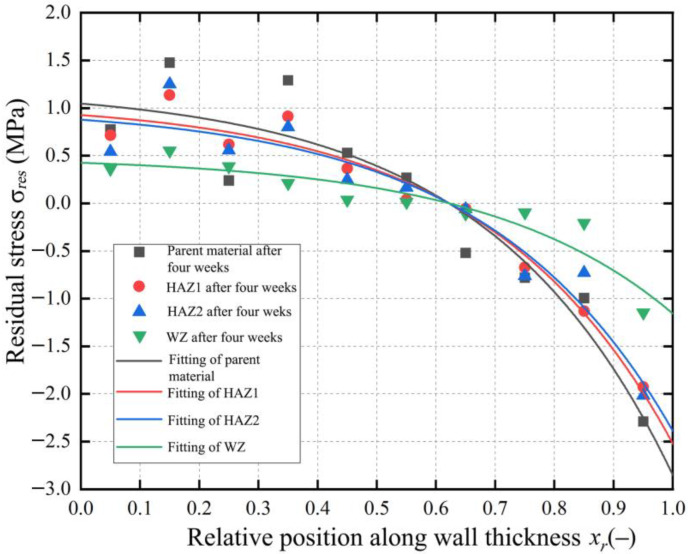
Residual stress distribution in each zone after four weeks.

To study the direct causes of the changes in the residual stress distribution in each joint zone relative to the BM, more detailed analysis of the crystallinity in each zone was conducted. Figure 16 shows the XRD patterns of different zones of the PE gas pipe. The pipe samples from each zone were divided into two parts along the wall thickness direction, which were divided into inner sample and outer sample. The crystallinity of each layer was measured using XRD testing, with the refined RWP values of the crystallinity being less than 10%, indicating sufficient accuracy in crystallinity calculations. The crystallinity values and the differences between the inner and outer wall thicknesses in each zone are summarized in Table 2. The crystallinity decreases progressively from the WZ to the HAZ and further to the BM zone, with HAZ1 and HAZ2 showing improved crystallinity compared to the BM after being affected by fusion heat. As shown in Figure 17, the degree of the residual stress reduction in the HAZs compared to the BM, and the more significant reduction in the WZ, corresponds to the crystallinity distribution pattern. The differences in crystallinity between the inner and outer layers within each zone are relatively small, ranging from 0.88% to 3.07%, indicating that the crystallization behavior is uniform across the pipe wall thickness. From the degree of the residual stress change in each zone as shown in Figure 17, it can be seen that the residual stress in the HAZ is lower compared to the BM zone, and the residual stress in the WZ reduces even further compared to the BM zone, which corresponds to the distribution pattern of crystallinity.

In each zone, the crystallinity of the outer layer of the pipe wall is lower than that of the inner layer, and the difference in crystallinity between the inner and outer layers increases from the WZ to the BM zone. The results of crystallinity in the different zones of inner and outer layer wall thickness can also explain why the residual stress on the outer wall of pipe is higher than that on the inner wall in the different zones. This is due to the faster cooling rate of the outer layer wall thickness of the pipe. The PE molecular chains rearrange during the heating process in the WZ zone and they interweave mutually and intertwine more tightly under the pressure fusion effect, which forms large dense network structure and increases the crystallinity.

Figure 17 shows the residual stress distribution in each zone after four weeks; the fluctuation in residual stress along the wall thickness in different zones can be explained by the difference in crystallinity between the inner and outer layer wall thickness. The WZ has a small difference in crystallinity between the inner and outer layer wall thickness and this zone has a smooth residual stress distribution curve. The HAZ2 has a smaller difference in crystallinity between the inner and outer layer wall thickness compared to HAZ1, but not by much, and also has a slightly smoother residual stress distribution curve compared to HAZ1. While the BM zone has the largest difference in crystallinity between the inner and outer layer wall thickness, the BM has a much greater fluctuation range of the residual stress distribution curve. The crystallinity distribution is consistent with the previously fitted residual stress distribution in each zone.

## 4. Conclusions

The paper studies the residual stresses distribution of butt fusion joints for PE gas pipes, and the following conclusions are drawn:

1. Based on a layer-by-layer ring cutting test method designed for accurately testing the circumferential residual stress in the PE pipe, the residual stress distribution was calculated by machining specific samples and measuring their diameter change. It was found that the residual stresses in the BM zone, the WZ, and the HAZ of the butt fusion joint all exhibit an exponential distribution along the pipe wall thickness. Analyzing the residual stress distribution results one week to four weeks later, the fluctuation ranges of the residual stress in all zones increased, indicating that the material gradually stabilized and the internal residual stress of the pipe was released.

2. The distribution of residual stress in various zones was analyzed, revealing that after butt fusion, the heat generated during the fusion process significantly affected the internal residual stress of the pipe. The residual stress in the WZ and heat-affected zone (HAZ1 and HAZ2) is improved compared to the BM zone. Moreover, the overall residual stress level of the butt fusion joint remains relatively low, indicating that the welding process does not introduce excessive internal stress into the material system.

3. The crystallinities of the WZ, HAZ, and BM zone were measured by using XRD technology. The crystallinity gradually decreases from the WZ to the HAZ and the BM zone. The crystallinity of the outer layer of the pipe wall in each zone is less than that of the inner layer, and the difference in crystallinity between the inner and outer layers increases from the WZ to the BM zone. In general, the difference in crystallinity between the inner and outer wall layers across all zones is relatively small, ranging from 0.88% to 3.07%, which reflects the uniformity of the crystalline structure formed during the fusion process.

4. The distribution of crystallinity is consistent with the residual stress distribution obtained from the tests. The BM zone has the largest difference in crystallinity between the inner and outer layer wall thickness, resulting in a much greater fluctuation range of the residual stress distribution curve. In contrast, the WZ has a smaller difference in crystallinity between the inner and outer layer wall thickness and has a smoother residual stress distribution curve. The difference in crystallinity between the inner and outer layer wall thickness of the HAZ is intermediate, and the residual stress distribution curve fluctuation is also intermediate.

## Figures and Tables

**Table 1:** Residual stress values on the inner and outer walls of each zone.

$\mathrm{Relative}\;\mathrm{Position}\;x_{r}$	Residual Stress (MPa)			
	WZ	HAZ2	HAZ1	BM
Inner wall $x_{r}=0$	0.42	0.88	0.93	1.05
Outer wall $x_{r}=1$	1.15	2.38	2.51	2.75

**Table 2:** Crystallinity and difference values of inner and outer wall thicknesses in each zone.

Wall Thickness Position	Crystallinity (%)			
	WZ	HAZ2	HAZ1	BM
Inner layer	79.87	76.44	70.31	70.24
Outer layer	78.99	74.47	68.14	67.17
Difference	0.88	1.97	2.17	3.07

## Data Availability

The data presented in this study are available on request from the corresponding author. The data is not publicly available.

## References

[1] Compa M., Alomar C., Mourre B., March D., Tintoré J., Deudero S. (2020). Nearshore spatio-temporal sea surface trawls of plastic debris in the Balearic Islands. Mar. Environ. Res..

[2] Nasiri S., Khosravani M.R. (2023). Failure and fracture in polyethylene pipes: Overview, prediction methods, and challenges. Eng. Fail. Anal..

[3] Anwar M.K., Shah S.A.R., Alhazmi H. (2021). Recycling and Utilization of Polymers for Road Construction Projects: An Application of the Circular Economy Concept. Polymers.

[4] Wang B., Li J. (2023). Quality Evaluation of Hot Melt Welding of Plastic Pipes. Acad. J. Sci. Technol..

[5] Novakovic B., Kashkoush M. (2023). Modeling the matching stage of HDPE hot plate welding: A study using regression and support vector machine models. Polym. Eng. Sci..

[6] Lai H., Fan D., Liu K. (2022). The Effect of Welding Defects on the Long-Term Performance of HDPE Pipes. Polymers.

[7] De A., DebRoy T. (2011). A perspective on residual stresses in welding. Sci. Technol. Weld. Join..

[8] Tabatabaeian A., Ghasemi A.R., Shokrieh M.M., Marzbanrad B., Baraheni M., Fotouhi M. (2021). Residual Stress in Engineering Materials: A Review. Adv. Eng. Mater..

[9] Navid Moslemi Abdi B., Gohari S., Izman Sudin Norizah Redzuan Amran Ayob Ahmed M., Rhee S., Burvill C. (2022). Influence of welding sequences on induced residual stress and distortion in pipes. Constr. Build. Mater..

[10] Kumar R., Mahapatra M.M., Pradhan A.K., Giri A., Pandey C. (2023). Experimental and numerical study on the distribution of temperature field and residual stress in a multi-pass welded tube joint of Inconel 617 alloy. Int. J. Press. Vessel. Pip..

[11] Biradar A., Moreno J., Mertiny P. Assessing residual stresses in PE-RT pipes and annealing temperature sensitivity analysis. Proceedings of the ASME 2023 Pressure Vessels & Piping Conference.

[12] Wang J., Huo L., Gu K. (2006). Finite-element analyses of instantaneous stresses of hot-plate welded joint of plastic pressure pipes. Trans. China Weld. Inst..

[13] Sun Y., Jia Y., Haroon M., Lai H., Jiang W., Tu S.-T. (2019). Welding Residual Stress in HDPE Pipes: Measurement and Numerical Simulation. J. Press. Vessel Technol.-Trans. ASME.

[14] Khademi Zahedi R., Alimouri P., Khademi Zahedi H., Shishesaz M. (2019). Investigating peak stresses in fitting and repair patches of buried polyethylene gas pipes. Front. Struct. Civ. Eng..

[15] Bai F., Ding H., Tong L., Pan L. (2020). Microstructure and properties of the interlayer heat-affected zone in X80 pipeline girth welds. Prog. Nat. Sci. Mater. Int..

[16] Xu S., Wang W. (2013). Numerical investigation on weld residual stresses in tube to tube sheet joint of a heat exchanger. Int. J. Press. Vessel. Pip..

[17] Kogo B.E. (2021). Thermal and residual stress analysis of welded joints in cladded pipelines. Doctoral Thesis.

[18] Starostin N.P., Tikhonov R.S., Nikolaeva M.A., Akimov M.P. (2024). Stress-Strain State of a Welded Joint in Welding a Saddle Branch to a Polyethylene Pipe under Low Temperatures. Proc. Southwest State Univ..

[19] Zhao W., Jiang W., Zhang H., Han B., Jin H., Gao Q. (2021). 3D finite element analysis and optimization of welding residual stress in the girth joints of X80 steel pipeline. J. Manuf. Process..

[20] Chapman T., Gillespie J., Pipes R., Månson J., Seferis J. (1990). Prediction of process-induced residual stresses in thermoplastic composites. J. Compos. Mater..

[21] Chason E., Karlson M., Colin J., Magnfält D., Sarakinos K., Abadias G. (2016). A kinetic model for stress generation in thin films grown from energetic vapor fluxes. J. Appl. Phys..

[22] Perić M., Nižetić S., Garašić I., Gubeljak N., Vuherer T., Tonković Z. (2020). Numerical calculation and experimental measurement of temperatures and welding residual stresses in a thick-walled T-joint structure. J. Therm. Anal. Calorim..

[23] Perić M., Garašić I., Gubeljak N., Tonković Z., Nižetić S., Osman K. (2022). Numerical Simulation and Experimental Measurement of Residual Stresses in a Thick-Walled Buried-Arc Welded Pipe Structure. Metals.

[24] Tan N., Lin L., Deng T., Dong Y. (2022). Evaluating the residual stress and its effect on the quasi-static stress in polyethylene pipes. Polymers.

[25] Wu C., Kim J.-W. (2018). Analysis of welding residual stress formation behavior during circumferential TIG welding of a pipe. Thin-Walled Struct..

[26] Majid S., Farahani M. (2019). Experimental Study of the Residual Stresses in Girth Weld of Natural Gas Transmission Pipeline. J. Appl. Comput. Mech..

[27] Xie Y., Zhuang J., Huang B., Chen Q., Li G. (2020). Effect of different welding parameters on residual stress and deformation of 20/0Cr18Ni9 dissimilar metal arc-welding joint. J. Adhes. Sci. Technol..

[28] Golikov N.I. (2020). Effect of Residual Stress on Crack Development in Longitudinal Welded Joints of a Gas Pipeline. Procedia Struct. Integr..

[29] Zhang H., Wang Y., Han T., Bao L., Wu Q., Gu S. (2020). Numerical and experimental investigation of the formation mechanism and the distribution of the welding residual stress induced by the hybrid laser arc welding of AH36 steel in a butt joint configuration. J. Manuf. Process..

[30] Craig R.R., Taleff E.M. (2020). Mechanics of Materials.

[31] Lomazov V.A., Lomazova V.I., Akupiyan O.A., Miroshnichenko I.V., Petrosov D.A. (2023). Mathematical modeling of diagnostics of residual stresses in a layered medium. J. Phys. Conf. Ser..

[32] Rahul Y., Vipindas K. (2021). Methodology for prediction of sub-surface residual stress in micro end milling of Ti-6Al-4V alloy. J. Manuf. Process..

[33] Bosire R.N., Muvengei O.M., Mutua J.M., Kimotho J.K. (2023). Finite element based model for predicting induced residual stresses and cutting forces in AISI 1020 steel alloy. Mater. Werkst..

[34] Clausen B., D’Elia C.R., Prime M.B., Hill M.R., Bishop J.E., Johnson K.L., Jared B.H., Allen K.M., Balch D.K., Roach R.A. (2020). Complementary Measurements of Residual Stresses Before and After Base Plate Removal in an Intricate Additively-Manufactured Stainless-Steel Valve Housing. Addit. Manuf..

[35] Poduška J., Kučera J., Hutař P., Ševčík M., Křivánek J., Sadílek J., Náhlík L. (2014). Residual stress distribution in extruded polypropylene pipes. Polym. Test..

[36] Poduška J., Hutař P., Kučera J., Frank A., Sadílek J., Pinter G., Náhlík L. (2016). Residual stress in polyethylene pipes. Polym. Test..

[37] Huang Z., Tang H., Ding Y., Wei Q., Xia G. (2017). Numerical Simulations of temperature for the in-service welding of gas pipeline. J. Mater. Process. Technol..

[38] Midawi A.R., Sherepenko O., Ramachandran D.C., Akbarian S., Shojaee M., Zhang T., Ghassemi-Armaki H., Worswick M., Biro E. (2023). Prediction of mechanical properties in the sub-critical heat affected zone of AHSS spot welds using Gleeble thermal simulator and Hollomon-Jaffe model. Metals.

